# Bromoform exposure is associated with non-melanoma skin cancer: evidence from NHANES 2011–2020

**DOI:** 10.3389/fpubh.2023.1191881

**Published:** 2023-10-19

**Authors:** Mingnan Gao, Han Guo, Jingjing Han, Jinhua Liu, Yinglong Hou, Zimao Wang, Zheng Yang, Qiying Wang

**Affiliations:** Department of Plastic Surgery, First Affiliated Hospital of Zhengzhou University, Zhengzhou University, Zhengzhou, China

**Keywords:** trihalomethanes, bromoform, non-melanoma skin cancer, water, swimming, cancer prevention

## Abstract

**Background:**

Non-melanoma skin cancer (NMSC) is a prevalent skin malignancy. It has been indicated in many studies that trihalomethanes (THMs) exposure has a strong association with tumors but has not been associated with NMSC. Our investigation aims to explore the association between THMs exposure and NMSC.

**Methods:**

Cross-sectional data from the 2011 to 2020 National Health and Nutrition Examination Survey (NHANES) was collected. Poisson regression and subgroup analyses were performed to evaluate the association between individual THMs components and NMSC. Fitted smoothing curves and generalized additive models were also used.

**Results:**

This study involved 5,715 individuals, 98 (1.7%) of whom self-reported NMSC. After adjusting for covariates, Poisson regression showed that higher blood TBM levels were associated with an increased likelihood of NMSC (OR = 1.03; 95% CI: 1.01–1.05, *p* = 0.002). However, the correlation between the blood levels of TCM, DBCM, and BDCM and the likelihood of NMSC was not statistically significant (all *p* > 0.05). Subgroup analysis and interaction tests showed no significant differences between blood TBM concentration and the likelihood of NMSC, indicating that age, gender, and race were significantly independent of this positive association (all *p* < 0.05).

**Conclusions:**

Our results implied that among adults older than 65 years old in the U.S., elevated blood TBM concentrations were positively associated with NMSC. More prospective investigations are required to validate this relationship with the early prevention of NMSC.

## Introduction

Non-melanoma skin cancer (NMSC) is a prevalent kind of skin malignancy that includes squamous and basal cell carcinoma of the skin (1). In recent years, the incidence of NMSC is increasing (2, 3). The high prevalence and rising incidence of treatment place a huge cost burden on patients and healthcare systems (4–7). By being more aware of the risk factors, it is possible to avoid NMSC and recognize it early, reducing its impact.

When chlorine used for disinfection interacts with organic or inorganic materials in water, disinfection by-products (DBPs) are produced, and trihalomethanes (THMs) are one of the significant DBPs produced. THMs contain chloroform (TCM), dibromochloromethane (DBCM), bromodichloromethane (BDCM), and bromoform (TBM). Even in minimal concentrations, these chemicals are detrimental to human health. Different cancers, reproductive issues, birth defects, and miscarriages are just a few examples of these health risks (8–11). THMs are thought to have a chance of causing cancer, according to studies (12). THMs were found to be present in drinking water after chlorination in 1972, and since then, research has been done to determine how they originate, how hazardous they are, how common they are, and how to reduce them (13, 14). TTHMs' maximum contaminant limit (MCL) was established at 100 μg/L (15). The MCL for TTHMST was decreased by The Stage 1 D-DBP Rule to 80 μg/L (16).

Tap water, also known as drinking water, is used for washing, cleaning, cooking, bathing, and other activities. As a result, THMs can be consumed and absorbed not only orally but also by inhalation exposure and absorption as well as through contact with the skin. Studies from various regions have produced varying conclusions regarding whether the exposure pathway carries the most risk of developing cancer (17–20). Lifetime carcinogenic risk assessment of different components of THMs under various exposure routes is mainly achieved by calculating chronic daily intake (CDI) and potency factor (PF) (8), and the product of these indicators is the lifetime carcinogenic risk of a THMs component under a certain exposure route. CDI is the mass of a substance per unit of body weight per unit of exposure time. A drug's PF calculates the lifetime cancer risk associated with exposure to that medicine. It is typically given as the percentage of the population affected per kilogram of body weight per day per milligram of substance. According to specific research that evaluated the carcinogenic risk of various THMs components (17, 18, 21, 22), TCM was the primary constituent of all THMs and the primary contributor to overall cancer risk. Other research found that BDCM had the highest percentage contribution (8, 23). A recent study in India showed that TBM had the most significant levels and concentrations and an enormous percentage contribution to overall cancer risk (20).

According to several research, THMs exposure is strongly associated with malignancies, including bladder, colorectal, and breast cancers (24–27). However, the association between NMSC and THMs has not been examined. Moreover, exposure evaluations relied on total THMs concentrations measured or calculated in THMs mixes, particularly in drinking water, rather than exposure levels of individual THMs components, in most research investigating the link between exposure to THMs and a specific tumor. In these circumstances, studies investigating the association of individual THMs components with the likelihood of NMSC would be valuable.

Therefore, we investigated general and representative U.S. populations using National Health and Nutrition Examination Survey (NHANES) data from 2011 to 2020 to investigate the association between total blood THMs levels and individual THMs components and NMSC.

## Subject and methods

### Data and sample sources

**Figure 1:**
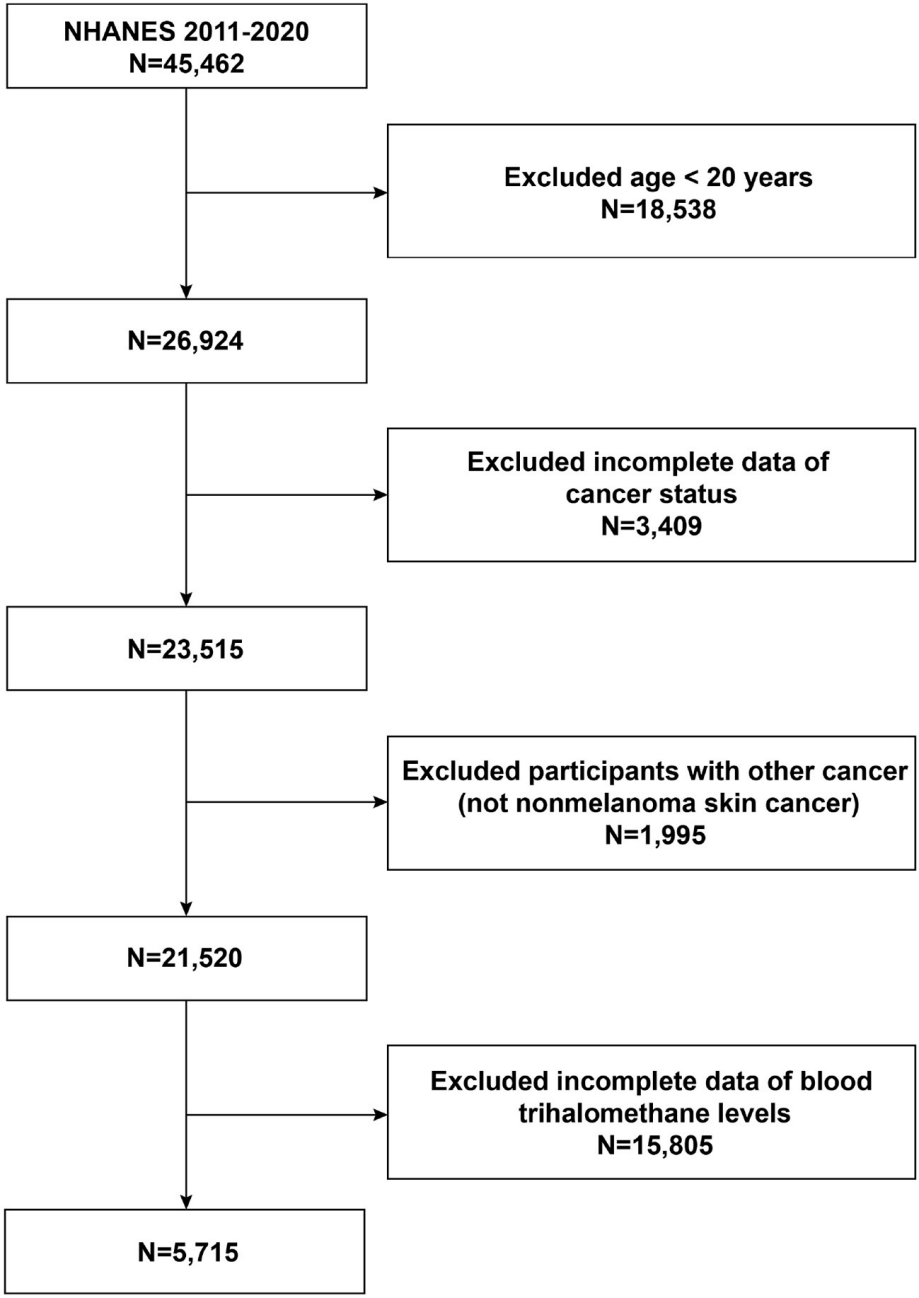
Flowchart of the participants' selection from NHANES 2011–2020.

Sample sources and techniques data were acquired from NHANES, a cross-sectional nationwide population-based survey carried out by the National Center for Health Statistics (NCHS) to collect details on potential health risk factors and the nutritional status of citizens in the United States. To gather a representative sample of the total American population, a sophisticated stratified, multistage probability whole-group sampling design was used in its creation (28). The NCHS Research Ethics Review Committee clarified to the study's protocol for the NHANES. All participants in the poll who were under the age of 16 provided their parents' or guardians' written informed consent. The detailed NHANES research design and data are accessible at https://www.cdc.gov/nchs/nhanes/. Participants completed standardized home interviews, health exams at mobile screening facilities, and lab tests to collect laboratory data to assess their physical and medical conditions. To examine the relationship between the elevated likelihood of NMSC and blood concentrations of THMs, we chose five NHANES cycles from 2011 to 2020. In our analysis, exclusion criteria for participants were (1) aged <20 years, (2) lack of data on cancer prevalence, (3) having cancer other than non-melanoma skin cancer, and (4) lack of data on blood concentrations of THMs. A total of 45,462 participants were initially recruited. After excluding participants age <20 years (*n* = 18,538), with missing cancer data (*n* = 3,409), with other cancers (*n* = 1,995), and with missing data on THMs (*n* = 15,805), in our final analysis, 5,715 eligible subjects with a minimum age of 20 were included. The specific patient screening flowchart is shown in Figure 1.

### Exposure variable

Throughout the test, venous blood samples were collected to assess the blood THMs levels. No additional conditions, such as fasting or specific diets, were necessary to collect blood samples. Whole blood samples were taken in clean 10 or 7 ml blood collection glass tubes, including anticoagulants with potassium oxalate and sodium fluoride. Those blood samples were prepared, kept, and sent to the National Center for Environmental Health, Centers for Disease Control and Prevention's Environmental Health Laboratory Sciences Division for analysis. Using headspace solid phase microextraction/gas chromatography/isotope dilution mass spectrometry (SPME/GC/isotope dilution MS), a similar method described by Blount (29), blood concentrations of TCM, DBCM, BDCM, and TBM were measured. This approach offers higher throughput, improved ruggedness, and lower costs for large-scale studies (29). With this method, levels of each component of THMs in the blood can be measured down to low parts per trillion. This method is useful for figuring out these amounts and looking into sustained or recent low-level exposure cases because non-occupationally exposed people have blood THMs concentrations in this range. Blood concentrations of TCM, DBCM, BDCM, and TBM, which were measured, were used to calculate the total THMs (sum of individual THMs concentrations) and total brominated THMs (sum of TBM, DBCM, and BDCM concentrations) in the blood (30). Laboratory analyses were subject to stringent quality control measures. Quality assurance and quality control procedures followed standard practices (31). Daily, the stability of the analytical system was tested experimentally. Each day's run sequence was supplemented with standards and quality control materials. In each run, the water blank and two QC samples at various concentrations were among the minimum three quality assessment sample types examined. These samples had all been created using unidentified blood samples. A water blank was prepared using the standards in addition to these samples. To confirm the effectiveness of the procedure and the instrument, absolute responses and their retention times from the lowest calibrator were analyzed from the previous run. Data that fell below the detection threshold was padded with an interpolated value. This value is the lower limit of detection divided by the square root of 2 (LLOD/sqrt [2]).

### Covariates

Demographic baseline data were obtained from 2011 to 2020 NHANES interview data and included: age, gender (male or female), race, education level, the ratio of family income to poverty (PIR), body mass index (BMI), and smoking status. We categorized race as white or non-white, with the latter category including Mexican American, other Hispanic, non-Hispanic black, and other races (32). For income, we used PIR, defined in NHANES as total household income divided by the federal poverty level. Based on the Supplemental Nutrition Assistance Program (SNAP) eligibility criteria cited in the NHANES Analysis Guide 1999–2010, we used the following income categories: 0–1.30 (the lowest income), 1.31–3.50 (the middle income), or 3.51–5.00 (the highest income) (33). We used the following education categories: ≤ high school graduation, some college or associate's degree, or ≥ college graduation (32). According to the question “Have you smoked at least 100 cigarettes in your lifetime?” smoking status was divided into two categories, never smoked or ever smoked.

### Statistical analyses

Using the proper NHANES sampling weights and considering intricate multistage whole-group surveys, all statistical analyses were carried out by the Centers for Disease Control and Prevention (CDC) recommendations. Continuous data were presented as means with standard errors (SE), while categorical variables were given as proportions. A *t*-test (for continuous variables) or chi-square test (for categorical variables) was used to assess differences between participants with NMSC and those without cancer. Three different models of the likelihood of NMSC were each subjected to Poisson regression analysis to examine the relationship between blood THMs levels and their various constituents. In model 1, no adjustment for covariates was made. In model 2, gender, age, and race were adjusted. Model 3 was adjusted for gender, age, race, education level, PIR, and smoking status. Age (20-34/35-49/50-64/≥65 years), gender (male/female), and race (white/non-white) stratified variables were used in subgroup analyses of the connection between blood THMs concentrations and the likelihood of NMSC. These stratification criteria were also considered as predetermined potential impact modifiers. An interaction term was included to check for heterogeneity of connections between subgroups. A *t*-test (for continuous variables) was also used to assess differences in the distribution of blood THMs concentrations between subgroups. NMSC and blood TBM concentrations were evaluated for nonlinear relationships using generalized additive models and smoothed curve fitting. All analyses were performed using PackageR (http://www.R-project.org), EmpowerStats (www.empowerstats.com), and Stata/MP V.17.0 (StataCorp). The statistically significant level was set as *p* < 0.05.

## Results

### Baseline characteristics of participants

Subject baseline characteristics included 5,715 subjects, 49.10% male and 50.90% female, with a mean age of 49.00 ± 17.05 years; 1.7% of participants were classified as NMSC patients. The distribution of age, gender, race, education level, PIR, smoking status, and blood TBM concentrations between those without cancer and those with NMSC showed statistically significant disparities (all *p* < 0.05). Subjects who were more likely to have NMSC were older age, men, white, lower education level, lower PIR, smokers, and higher blood TBM levels in our study (all *p* < 0.05). Table 1 displays the participants' clinical and biochemical characteristics based on NMSC.

**Table 1:** Characteristics of the study population based on NMSC^a^ in the NHANES^b^ 2011–2020.

**Characteristics**	**Overall (*N* = 5,715)**	**Non-NMSC (*N* = 5,617)**	**NMSC (*N* = 98)**	***p*-value^c^**
Age, mean ± SD^d^ (years)	49.00 ± 17.05	48.66 ± 16.93	68.09 ± 12.53	<0.001
**Gender**, ***n*** **(%)**				0.044
Male	2,806 (49.10)	2,748 (48.92)	58 (59.18)	
Female	2,909 (50.90)	2,869 (51.08)	40 (40.82)	
**Race**, ***n*** **(%)**				<0.001
White	2,089 (36.55)	1,998 (35.57)	91 (92.86)	
Non-white^e^	3626 (63.45)	3,619 (64.43)	7 (7.14)	
**Education level**, ***n*** **(%)**				<0.001
≤ High school graduate	2,506 (43.85)	2,482 (44.19)	24 (24.49)	
Some college or associate's degree	1,782 (31.18)	1,747 (31.10)	35 (35.71)	
≥College graduate	1,425 (24.93)	1,386 (24.68)	39 (39.80)	
Missing	2 (0.03)	2 (0.04)	0 (0.00)	
**PIR**^**f**^, ***n*** **(%)**				<0.001
0–1.30	1,553 (27.17)	1,542 (27.45)	11 (11.22)	
1.31–3.50	1,874 (32.79)	1,840 (32.76)	34 (34.69)	
3.51–5.00	1,618 (28.31)	1,575 (28.04)	43 (43.88)	
Missing	670 (11.72)	660 (11.75)	10 (10.20)	
BMI^g^, mean ± SD (kg/m^2^)	29.54 ± 7.19	29.54 ± 7.21	29.00 ± 6.25	0.460
**Smoking status**, ***n*** **(%)**				0.010
Ever	2,367 (41.42)	2314 (41.20)	53 (54.08)	
Never	3,348 (58.58)	3303 (58.80)	45 (45.92)	
Bromoform, mean ± SD (pg/mL)	14.00 ± 21.02	6.48 ± 4.33	7.56 ± 6.77	0.016
Bromodichloromethane, mean ± SD (pg/mL)	5.03 ± 3.22	5.03 ± 3.21	5.26 ± 3.88	0.484
Dibromochloromethane, mean ± SD (pg/mL)	4.22 ± 2.59	4.42 ± 2.58	4.63 ± 52.86	0.420
Chloroform, mean ± SD (pg/mL)	6.50 ± 4.39	14.00 ± 21.14	13.81 ± 13.11	0.928
Total THMs, mean ± SD (pg/mL)	29.95 ± 23.56	29.93 ± 23.64	31.26 ± 18.82	0.580
Total brominated THMs, mean ± SD (pg/mL)	15.96 ± 7.61	15.93 ± 7.56	17.45 ± 10.28	0.050

a NMSC, non-melanoma skin cancer.

b NHANES, National Health and Nutrition Examination Survey.

c *p*-values were calculated using the *t*-test for binomial groups and the Chi-square test for categorical groups.

d Mean ± SD for continuous variables.

e Includes Mexican American, other Hispanic, non-Hispanic Black, and other races.

f PIR, the ratio of family income to poverty. On the basis of the Supplemental Nutrition Assistance Program eligibility criteria cited in NHANES analytic guidelines 1999–2010, 0–1.30 indicates lowest income, 1.31–3.50 indicates middle income, and 3.51–5.00 indicates highest income (33).

g BMI, body mass index.

### The association between blood TBM concentrations and NMSC

Our results suggested that, based on current data, elevated blood TBM concentrations were correlated with the likelihood of NMSC. This correlation was significant in our crude model (OR = 1.02; 95% CI: 1.00–1.04, *p* = 0.027) and the minimally adjusted model (OR = 1.03; 95% CI: 1.01–1.05, *p* = 0.006). The positive correlation between blood TBM concentrations and the likelihood of NMSC persisted in the fully adjusted model (OR = 1.03; 95% CI: 1.01–1.05, *p* = 0.002). However, the correlation between the blood levels of TCM, DBCM, and BDCM and the likelihood of NMSC was not statistically significant in either the crude model, the minimally adjusted model, or the fully adjusted model (all *p* > 0.05). So, it could not be inferred that the blood levels of TCM, BDCM, and DBCM were related to the likelihood of NMSC (Table 2).

**Table 2:** The association between blood concentrations of individual THMs components and NMSC^a^.

	**OR**^**b**^ **(95%CI)**^**c**^, ***p*****-value**		
	**Crude model (model 1)** ^d^	**Minimally adjusted mode (model 2)** ^e^	**Fully adjusted model (model 3)** ^f^
**Bromoform, pg/mL**	1.02 (1.00, 1.04)	1.03 (1.01, 1.05)	1.03 (1.01, 1.05)
	0.027	0.006	0.002
**Bromodichloromethane, pg/mL**	1.02 (0.97, 1.07)	1.02 (0.99, 1.06)	1.02 (0.98, 1.05)
	0.487	0.250	0.470
**Dibromochloromethane, pg/mL**	1.03 (0.97, 1.09)	1.05 (0.99, 1.11)	1.05 (0.99, 1.11)
	0.425	0.089	0.079
**Chloroform, pg/mL**	1.00 (0.99, 1.01)	1.00 (0.00, 1.01)	1.00 (0.99, 1.01)
	0.929	0.883	0.945

a THMs, trihalomethanes; NMSC, non-melanoma skin cancer.

b OR: odds ratio.

c 95% CI: 95% confidence interval.

d Model 1: no covariates were adjusted.

e Model 2: adjusted for gender, age, and race.

f Model 3: adjusted for gender, age, race, education level, PIR.

### Subgroup analysis

**Figure 2:**
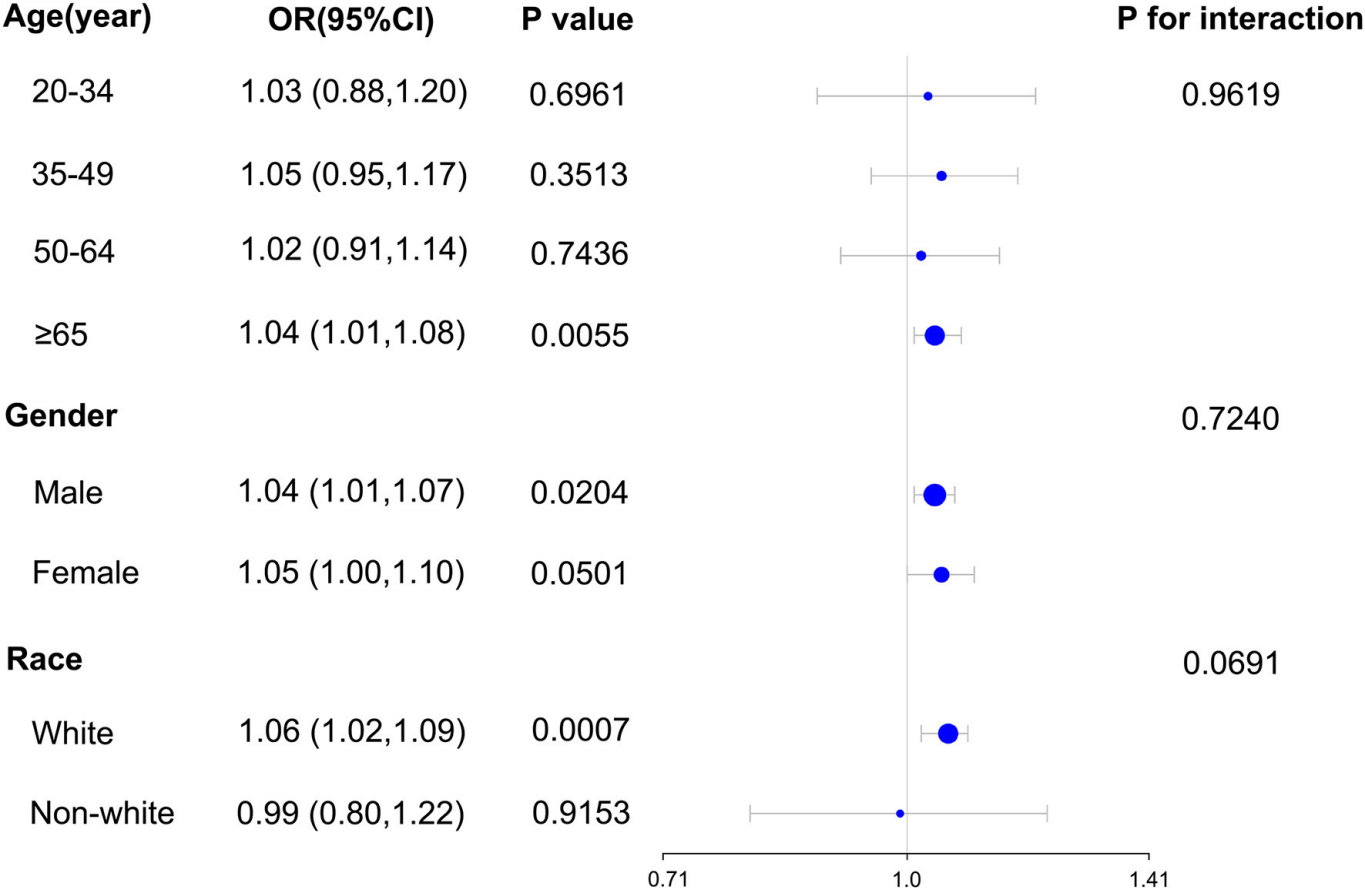
Subgroup analysis for the association between blood TBM concentrations and NMSC.

Our subgroup analysis's findings revealed a significant relationship between blood TBM concentrations and the likelihood of NMSC in the population age ≥ 65 (OR = 1.04; 95% CI: 1.01–1.08, *p* = 0.0055) in subgroups stratified by age. For subgroups stratified by gender, blood TBM concentrations and the likelihood of developing NMSC were shown to be significantly correlated in the male population (OR = 1.04; 95% CI: 1.01–1.07, *p* = 0.0204). In subgroups stratified by race, a significant association between blood TBM concentrations and the likelihood of NMSC was detected in the white racial population (non-Hispanic white; OR = 1.06; 95% CI: 1.02–1.09, *p* = 0.0007). Interaction tests revealed that the correlation between blood TBM concentrations and the likelihood of NMSC was not statistically different across each stratum, indicating that age, gender, and race did not substantially depend on this beneficial link (*p* > 0.05 for all interaction tests). At the same time, our findings suggest a stronger positive association between blood TBM concentrations and the likelihood of NMSC in older (≥65 years), male, and white individuals, although the interaction tests were insignificant (Figure 2). It was shown in Table 3 that there were statistically significant differences in age and race across the spectrum of blood TBM, DBCM, and TCM concentrations (all *p* < 0.05). Only the distribution of blood BDCM concentrations in the various age subgroups showed statistically significant differences (*p* < 0.001).

**Table 3:** The distribution of individual blood THMs^a^ concentrations in subgroups.

	**Bromoform (pg/mL)**	**Bromodichloromethane (pg/mL)**	**Dibromochloromethane (pg/mL)**	**Chloroform (pg/mL)**
**Age**				
20–34	6.73 ± 6.20	5.00 ± 2.90	4.42 ± 2.67	13.66 ± 18.99
35–49	6.43 ± 3.27	5.27 ± 3.75	4.56 ± 3.06	14.35 ± 22.96
50–64	6.35 ± 2.99	4.97 ± 3.05	4.38 ± 2.27	14.78 ± 24.68
≥65	6.51 ± 4.54	4.86 ± 3.08	4.32 ± 2.22	12.94 ± 14.58
*p*-value	0.039	<0.001	0.043	0.049
**Gender**				
Male	6.59 ± 5.10	5.10 ± 3.44	4.48 ± 2.71	13.70 ± 21.23
Female	6.41 ± 3.56	4.97 ± 3.00	4.37 ± 2.46	14.29 ± 20.82
*p*-value	0.486	0.811	0.681	0.08
**Race**				
White	6.47 ± 3.79	4.94 ± 3.39	4.32 ± 2.57	14.27 ± 25.95
Non-white	6.51 ± 4.70	5.09 ± 3.12	4.48 ± 2.60	13.84 ± 17.58
*p*-value	<0.001	0.113	<0.001	<0.001

a THMs, trihalomethanes.

### Smooth curve fitting

**Figure 3:**
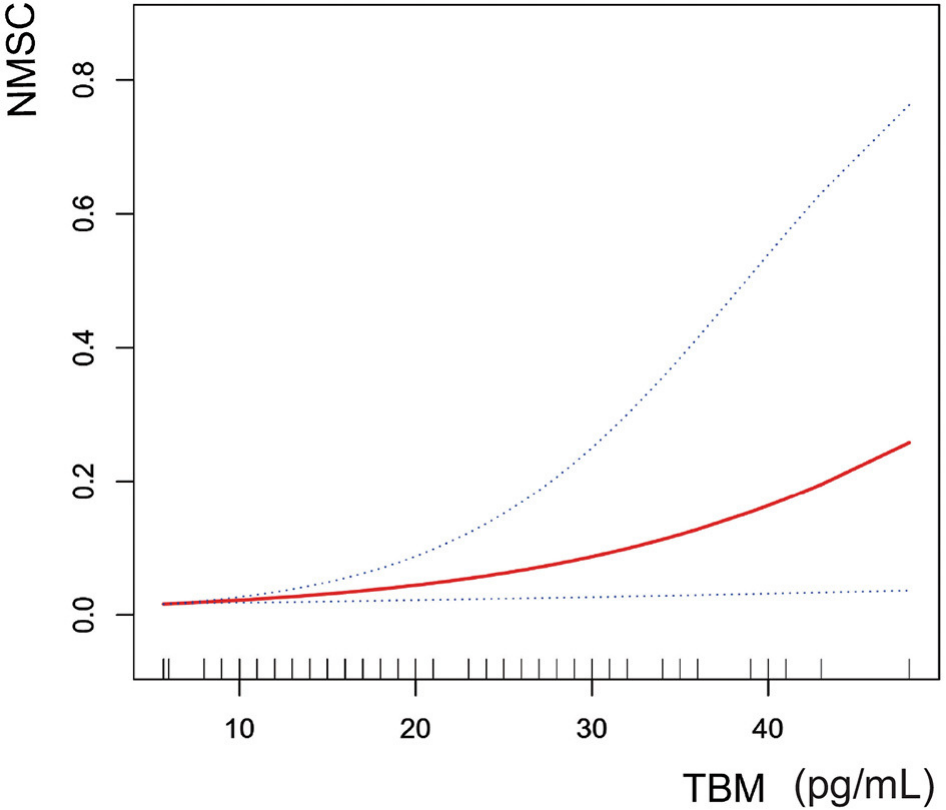
The correlation between blood TBM concentrations and NMSC (using penalized spline method). Age, gender, race, education level, PIR, and smoking status were adjusted.

We fitted the smoothed curve and used the generalized additive model to define the nonlinear association between blood TBM concentrations and the likelihood of NMSC. We excluded significant outliers with blood TBM concentrations >50 pg/mL. The results showed an increasing trend of smoothed fitted curves for the association of blood TBM concentrations in the range of 5.7–48 pg/mL with the likelihood of NMSC (Figure 3).

## Discussion

Our cross-sectional study that included 5,715 participants showed that blood TBM levels were significantly connected with the likelihood of NMSC in adults aged 65 and over. The results of interaction tests and subgroup analyses indicated that age, gender, and race did not substantially depend on this link. According to our research, increased blood TBM levels were an independent risk factor for NMSC.

Our results supported earlier research suggesting that exposure to THMs may impact NMSC development (34). For those with THMs levels >40 g/L, Karagas claimed that the OR for basal cell carcinoma was 2.4 (95% CI: 0.9–6.7). For squamous cell carcinoma, it was 2.1 (95% CI: 0.7–7.0) among people who reported using a public water supply system. There are presently just a few epidemiologic studies that show a connection between THMs exposure and nonmelanoma skin malignancies. However, there is some evidence for the link between THMs exposure and other malignancies. According to several epidemiological studies, THMs levels in water have been linked to several cancers, including bladder and colon cancers (35–38). Unfortunately, not all of these research have provided experimental proof that THMs induction causes cancer (35–37). Bladder cancer incidence and mortality were significantly positively correlated with exposure to THMs in tap water or swimming pools, according to multicenter case-control studies (39, 40). In addition, people with high levels of THMs had higher odds of developing colon cancer than people with low levels of THMs (41, 42). Jones et al. (43) published in 2019 the risk of rectal cancer with ingested total THMs (HR Q5 vs. Q1 = 1.71; 95% CI: 1.00–2.92), bromodichloromethane (HR Q4 vs. Q1 = 1.89; 95% CI: 1.17–3.00), and trichloroacetic acid (HR Q4 vs. Q1 = 1.92; 95% CI: 1.20–3.09) positive correlation between exposure estimates, but not for colon cancer. Most research concentrated on the association between total THMs exposure and cancer. Still, others, like ours, also paid attention to the associations between certain THMs components and cancer on their own. Instead of analyzing blood THMs levels, Bove et al. (44, 45) attempted to demonstrate the cancer risk linked to specific THMs exposure from drinking water. In these studies, TBM levels derived from water drinking strongly correlated with the risk of bladder cancer (OR = 3.05; 95 % CI: 1.51–5.69) and rectal cancer (OR = 1.85; 95 % CI: 1.25–2.74) in adult men. This is in line with the findings of our investigation. According to the results of our investigation, blood TBM levels were positively correlated with the likelihood of NMSC.

The main influencing factors explored in this study were THMs and their different components (TCM, DBCM, BDCM, and TBM), which are components of disinfection by-products (DBPs) produced during water treatment and linked to a higher risk of cancer in people (41, 46). THMs are the most prevalent category of DBPs (47, 48), and they have recently been the focus of epidemiological investigations. Several epidemiological studies showed that exposure to THMs had been linked to an increased risk of breast, colon, leukemia, gastric, and rectal cancers (24–27). The U.S. Environmental Protection Agency (EPA) has placed TCM in category B1 (probable human carcinogens with limited human data), BDCM and TBM in category B2 (probable human carcinogens with sufficient animal data), and DBCM in category C (probable human carcinogens). In addition to THMs, haloacetic acids (HAAs), and halo ketones (HKs) were also the most common forms of DBPs (47, 49–51). These include dichloroacetic acid (DCA), categorized as a B2 carcinogen, and trichloroacetic acid (TCA), categorized as a C carcinogen. TCA had been proven to cause chromosomal abnormalities in cells in several studies (52), and 1,1-Dichloropropanone had been shown to lower glutathione levels in cells (53). The permeability of THMs was around ten times greater than that of HKs, whereas the permeability of HAAs through the skin was extremely low. THMs are thus the most crucial risk factor when considering the danger of cutaneous exposure to DBPs (54). This evidence was another aspect that led us to choose THMs and the various parts that make them up as influencing factors for the study. Our results in the present study were comparable. Following the discovery that blood TBM concentrations were substantially related to the likelihood of NMSC in persons older than 20 years old by Poisson regression analysis: every 3% increase in the likelihood of NMSC was linked to every 1 pg/mL increase in blood TBM levels.

We hypothesized that direct dermal contact was a significant exposure mechanism for THMs connected to NMSC in terms of THMs exposure pathways. THMs can be exposed through various means, such as oral ingestion, inhalation, and direct skin contact. According to certain reports, inhalation was the primary exposure route (18), while the oral route was the most frequent (55–57). Given that THMs could enter the body through swimming, doing dishes, and coming into direct touch with chlorine-treated water while handling water (58), as well as the fact that there was direct contact dermatitis, we hypothesized that dermal exposure should also play a significant role in the exposure mechanism of NMSC. Several studies indicated that inhalation and cutaneous contact during swimming resulted in higher levels of THMs in the blood compared with oral exposure from drinking (59–61); some investigations revealed that cutaneous contact consumption of TCM was comparable to that caused by breathing (62). These studies provided evidence supporting our hypothesis that direct cutaneous contact was a significant exposure mechanism. Experiments by Xu et al. (54) regarding the skin's permeability to various THMs components revealed that TBM was the most permeable in the same state, which was also compatible with our findings.

Regarding the biological rationale for the interaction of THMs with NMSC, it had been pointed out that several genes that convert DBPs into reactive intermediates (CYP2E1 and GSTT1) were expressed in the skin and had a role in hereditary skin cancer susceptibility (63). Testing of TBM, DBCM, and BDCM revealed cytotoxic, genotoxic, and mutagenic effects in various experimental settings (including human cells) (64–79). The genotoxic and mutagenic properties of TBM, DBCM, and BDCM had been linked to the glutathione transferase family's genes, specifically GSTT1-1 (64, 80, 81). TBM, DBCM, and BDCM were converted by glutathione S-transferases (GST), which could then interact with DNA through the shift of the base pairs from GC to AT to produce mutations. Owing to their biological action, brominated THMs intermediates increased the likelihood of tumor growth by causing cellular dysregulation (64, 82–84). Several metabolic pathways were also implicated. Due to metabolic heterogeneity in their detoxification pathway, a family of cytochrome P450 (CYP) polymorphic variations, for instance, influenced the toxicity of brominated THMs. Furthermore, according to Ross and Pegram (71), the cell types implicated (liver and kidney cells) and the presence of the polymorphic variant CYP2E1 impacted how genotoxicity was assessed in an experimental rat model. This observation supported the hypothesis that the relative toxicity of these chemicals might be affected by polymorphic variations of enzymes engaged in detoxification processes. Moreover, sister chromatid exchanges, chromosomal abnormalities, and the development of micronuclei had all been linked to the mutagenic effects of DBCM and TBM in both animal and human cells (65, 66, 80, 85–87). According to current scientific research, the toxicity of THMs might be significantly influenced by brominated THMs (TBM, DBCM, and BDCM). Several investigations demonstrated that brominated THMs species had more potential for harm than TCM (64–67). TBM was one of the substances with the highest potential for mutagenesis and cytotoxicity (64, 87, 88). The strong mutagenic and cytotoxic potential of TBM further supported the findings of our investigation.

We made the following hypotheses regarding the finding that only TBM was connected with NMSC in this study. The permeability coefficients of THMs ranged from 0.16 to 0.21 cm/h when the donor solution was at 25°C, the ideal temperature for the human body, with TBM having the highest Kp value (54). Comparatively to the other THMs components, TBM was absorbed primarily through the skin. Second, numerous studies have revealed that brominated THM species had a higher potential for harm regarding cytotoxicity (74–77). One of the chemicals with the highest potential for mutagenicity and cytotoxicity was TBM (74, 85, 89).

There are a few restrictions on our study. First, we started by using NMSC's self-reported history. Because pathology or medical records didn't support it, misclassifying the results could produce bias. Although NHANES did not have an option for outcome validation, a validation study of a hospital cohort of almost 300 patients revealed that 92% of NMSC diagnoses were supported by patients' self-reported histories of skin cancer (90). Second, exposure frequency and duration significantly influence the correlation between TBM exposure and cancer. Nevertheless, because of the cross-sectional study design, we were unable to determine the frequency and duration of TBM exposure and the environmental TBM concentrations needed to calculate a chronic daily intake (CDI). As a result, we could not demonstrate a relationship between the likelihood of TBM exposure to NMSC over time. Due to the cross-sectional study design, we could not establish a direct causal link between TBM exposure and NMSC. Third, the relationship between lower exposure levels and the likelihood of NMSC could not be further investigated because the testing methods for determining the level of THMs and their components in blood have specific detection limits that are not sensitive to lower exposure levels. Additionally, the smoothed fit curve of blood TBM concentrations in the range of 5.7–48 pg/mL with the likelihood of NMSC showed an increasing trend after excluding the outliers in this study, making it impossible for us to determine the minimum pathogenic concentration to determine a specific optimal concentration control range. Nor can we speculate whether the effect of this risk factor on the likelihood of NMSC would plateau at higher levels of TBM exposure. That is to say, when all other factors are controlled for, the likelihood of NMSC does not rise once again when the blood TBM concentration reaches a specific higher value, and the concentration rises once more. They are crucial for furthering our understanding of the association between TBM exposure and the likelihood of NMSC. Therefore, future studies still need more participants and precise measurements to determine the causal relationship.

Notwithstanding these drawbacks, our study has several advantages. First and foremost, because we used a nationally representative group, our results can be broadly generalized. Secondly, our cohort's size allowed us to undertake subgroup analyses of blood TBM concentrations and the likelihood of NMSC by age, gender, and race. Third, we measured blood TBM concentrations rather than industry or occupation as a proxy for TBM exposure.

## Conclusion

Our study found that elevated blood TBM concentrations were positively associated with the likelihood of NMSC among adults aged 65 years and older. This may have preventive and diagnostic implications in clinical practice. Further, in-depth prospective studies are still needed to support our findings.

## Data availability statement

Publicly available datasets were analyzed in this study. This data can be found at: https://www.cdc.gov/nchs/nhanes/.

## Ethics statement

The studies involving human participants were reviewed and approved by the Research Ethics Review Board of the NCHS. Written informed consent to participate in this study was provided by the participants' legal guardian/next of kin.

## Author contributions

MG: data analysis and writing—original draft. HG: software. JH and JL: methodology. YH and ZW: conceptualization. ZY: writing—reviewing. QW: editing. All authors contributed to the article and approved the submitted version.

## References

[1] Miller DL Weinstock MA. Nonmelanoma skin cancer in the United States: incidence. J Am Acad Dermatol. (1994) 30:774–8. 10.1016/S0190-9622(08)81509-5 8176018

[2] Lomas A Leonardi-Bee J Bath-Hextall F. A systematic review of worldwide incidence of nonmelanoma skin cancer. Br J Dermatol. (2012) 166:1069–80. 10.1111/j.1365-2133.2012.10830.x 22251204

[3] Edwards BK Noone AM Mariotto AB Simard EP Boscoe FP Henley SJ. Annual report to the Nation on the status of cancer, 1975-2010, featuring prevalence of comorbidity and impact on survival among persons with lung, colorectal, breast, or prostate cancer. Cancer. (2014) 120:1290–314. 10.1002/cncr.28509 24343171 PMC3999205

[4] Housman TS Feldman SR Williford PM Fleischer AB Jr Goldman ND Acostamadiedo JM. Skin cancer is among the most costly of all cancers to treat for the Medicare population. J Am Acad Dermatol. (2003) 48:425–9. 10.1067/mjd.2003.186 12637924

[5] Chen JG Fleischer AB Jr Smith ED Kancler C Goldman ND Williford PM. Cost of nonmelanoma skin cancer treatment in the United States. Dermatol Surg. (2001) 27:1035–8. 10.1097/00042728-200112000-00010 11849266

[6] John Chen G Yelverton CB Polisetty SS Housman TS Williford PM Teuschler HV. Treatment patterns and cost of nonmelanoma skin cancer management. Dermatol Surg. (2006) 32:1266–71. 10.1097/00042728-200610000-00008 17034377

[7] Mudigonda T Pearce DJ Yentzer BA Williford P Feldman SR. The economic impact of non-melanoma skin cancer: a review. J Natl Comprehens Cancer Netw. (2010) 8:888–96. 10.6004/jnccn.2010.0066 20870635

[8] Mishaqa ESI Radwan EK Ibrahim M Hegazy TA Ibrahim MS. Multi-exposure human health risks assessment of trihalomethanes in drinking water of Egypt. Environ Res. (2022) 207:112643. 10.1016/j.envres.2021.112643 34973941

[9] Hwang BF Jaakkola JJ. Risk of stillbirth in the relation to water disinfection by-products: a population-based case-control study in Taiwan. PLoS ONE. (2012) 7:e33949. 10.1371/journal.pone.0033949 22457804 PMC3311556

[10] McDonald TA Komulainen H. Carcinogenicity of the chlorination disinfection by-product MX. J Environ Sci Health Part C. (2005) 23:163–214. 10.1080/10590500500234988 16291527

[11] Ewaid SH Rabee AM Al-Naseri SK. Carcinogenic risk assessment of trihalomethanes in major drinking water sources of Baghdad City. Water Resour. (2018) 45:803–12. 10.1134/S0097807818050202

[12] Bessonneau V Derbez M Clément M Thomas O. Determinants of chlorination by-products in indoor swimming pools. Int J Hyg Environ Health. (2011) 215:76–85. 10.1016/j.ijheh.2011.07.009 21862402

[13] Hamid A Riaz A Mohsin A. A review paper on disinfection by-products formation during drinking water treatment.

[14] Wang Z Li M Liao Y Pan Y Shuang C Li J. Formation of disinfection byproducts from chlorinated soluble microbial products: effect of carbon sources in wastewater denitrification processes. Chem Eng J. (2022) 432:134237. 10.1016/j.cej.2021.134237

[15] Li RA McDonald JA Sathasivan A Khan SJ. A multivariate Bayesian network analysis of water quality factors influencing trihalomethanes formation in drinking water distribution systems. Water Res. (2021) 190:116712. 10.1016/j.watres.2020.116712 33310438

[16] Chowdhury S Chowdhury IR Mazumder MAJ Al-Suwaiyan MS. Predicting risk and loss of disability-adjusted life years (DALY) from selected disinfection byproducts in multiple water supply sources in Saudi Arabia. Sci Tot Environ. (2020) 737:140296. 10.1016/j.scitotenv.2020.140296 32783866

[17] Mahato JK Gupta S. Advanced oxidation of Trihalomethane (THMs) precursors and season-wise multi-pathway human carcinogenic risk assessment in Indian drinking water supplies. Process Saf Environ Protect. (2022) 159:996–1007. 10.1016/j.psep.2022.01.066

[18] Tafesse N Porcelli M Hirpessa BB Gasana J Padhi R Garie SR. Exposure and carcinogenic risk assessment of trihalomethanes (THMs) for water supply consumers in Addis Ababa, Ethiopia. Toxicol Rep. (2023) 10:261–8. 10.1016/j.toxrep.2023.02.004 36876027 PMC9976571

[19] Anchal P Kumari M Gupta SK. Human health risk estimation and predictive modeling of halogenated disinfection by-products (chloroform) in swimming pool waters: a case study of Dhanbad, Jharkhand, India. J Environ Health Sci Eng. (2020) 18:1595–605. 10.1007/s40201-020-00578-6 33312664 PMC7721849

[20] Basu M Gupta SK Singh G Mukhopadhyay U. Multi-route risk assessment from trihalomethanes in drinking water supplies. Environ Monit Assess. (2011) 178:121–34. 10.1007/s10661-010-1677-z 20824332

[21] Hsu CH Jeng WL Chang RM Chien LC Han BC. Estimation of potential lifetime cancer risks for trihalomethanes from consuming chlorinated drinking water in Taiwan. Environ Res. (2001) 85:77–82. 10.1006/enrs.2000.4102 11161657

[22] Lahey W Connor M. The case for ocean waste-disposal. Technol Rev. (1983) 86:60–8.

[23] Lee S Guo H Lam S Lau S. Multipathway risk assessment on disinfection by-products of drinking water in Hong Kong. Environ Res. (2004) 94:47–56. 10.1016/S0013-9351(03)00067-7 14643286

[24] Costet N Villanueva C Jaakkola J Kogevinas M Cantor K King W. Water disinfection by-products and bladder cancer: is there a European specificity? A pooled and meta-analysis of European case-control studies. Occup Environ Med. (2011) 68:379–85. 10.1136/oem.2010.062703 21389011

[25] Villanueva CM Cantor KP Cordier S Jaakkola JJ King WD Lynch CF. Disinfection byproducts and bladder cancer: a pooled analysis. Epidemiology. (2004) 15:357–67. 10.1097/01.ede.0000121380.02594.fc 15097021

[26] Rahman MB Driscoll T Cowie C Armstrong BK. Disinfection by-products in drinking water and colorectal cancer: a meta-analysis. Int J Epidemiol. (2010) 39:733–45. 10.1093/ije/dyp371 20139236

[27] Font-Ribera L Gràcia-Lavedan E Aragonés N Pérez-Gómez B Pollán M Amiano P. Long-term exposure to trihalomethanes in drinking water and breast cancer in the Spanish multicase-control study on cancer (MCC-SPAIN). Environ Int. (2018) 112:227–34. 10.1016/j.envint.2017.12.031 29289867

[28] Curtin LR Mohadjer LK Dohrmann SM Kruszon-Moran D Mirel LB Carroll MD. National health and nutrition examination survey: sample design, 2007-2010. Vital Health Stat Series 2. (2013) 1–23. 25090039

[29] Blount BC Kobelski RJ McElprang DO Ashley DL Morrow JC Chambers DM. Quantification of 31 volatile organic compounds in whole blood using solid-phase microextraction and gas chromatography-mass spectrometry. J Chromatogr B. (2006) 832:292–301. 10.1016/j.jchromb.2006.01.019 16495163

[30] Min JY Min KB. Blood trihalomethane levels and the risk of total cancer mortality in US adults. Environ Pollut. (2016) 212:90–6. 10.1016/j.envpol.2016.01.047 26840521

[31] Taylor J. Quality Assurance of Chemical Measurements. Chelsea, MI: Lewis Publishing Company, Inc. (1987).

[32] Bedaiwi A Wysong A Rogan EG Clarey D Arcari CM. Arsenic exposure and melanoma among US adults aged 20 or older, 2003-2016. Publ Health Rep. (2022) 137:548–56. 10.1177/00333549211008886 33971104 PMC9109530

[33] Johnson CL Paulose-Ram R Ogden CL Carroll MD Kruszan-Moran D Dohrmann SM. National health and nutrition examination survey. Analytic guidelines, 1999-2010. (2013). 25090154

[34] Karagas MR Villanueva CM Nieuwenhuijsen M Weisel CP Cantor KP Kogevinas M. Disinfection byproducts in drinking water and skin cancer? A hypothesis. Cancer Causes Control. (2008) 19:547–8. 10.1007/s10552-008-9116-y 18219581 PMC2640837

[35] Kasim K Levallois P Johnson KC Abdous B Auger P. Chlorination disinfection by-products in drinking water and the risk of adult leukemia in Canada. Am J Epidemiol. (2006) 163:116–26. 10.1093/aje/kwj020 16319293

[36] Kuo HW Tiao MM Wu TN Yang CY. Trihalomethanes in drinking water and the risk of death from colon cancer in Taiwan. J Toxicol Environ Health Part A. (2009) 72:1217–22. 10.1080/15287390903129176 20077190

[37] Michaud DS Kogevinas M Cantor KP Villanueva CM Garcia-Closas M Rothman N. Total fluid and water consumption and the joint effect of exposure to disinfection by-products on risk of bladder cancer. Environ Health Perspect. (2007) 115:1569–72. 10.1289/ehp.10281 18007986 PMC2072844

[38] IARC Working Group on the Evaluation of Carcinogenic Risks to Humans. Chlorinated drinking-water; chlorination by-products; some other halogenated compounds; cobalt and cobalt compounds. IARC Monogr Eval Carcinog Risks Hum. (1991) 52:450. PMC7681469 1683674

[39] Chang CC Ho SC Wang LY Yang CY. Bladder cancer in Taiwan: relationship to trihalomethane concentrations present in drinking-water supplies. J Toxicol Environ Health Part A. (2007) 70:1752–7. 10.1080/15287390701459031 17885932

[40] Villanueva CM Cantor KP Grimalt JO Malats N Silverman D Tardon A. Bladder cancer and exposure to water disinfection by-products through ingestion, bathing, showering, and swimming in pools. Am J Epidemiol. (2007) 165:148–56. 10.1093/aje/kwj364 17079692

[41] King WD Marrett LD Woolcott CG. Case-control study of colon and rectal cancers and chlorination by-products in treated water. Cancer Epidemiol Biomark Prevent. (2000) 9:813–8. 10952098

[42] Kuo HW Tiao MM Tsai SS Wu TN Yang CY. Does calcium in drinking water modify the association between trihalomethanes and the risk of death from colon cancer? J Toxicol Environ Health Part A. (2010) 73:657–68. 10.1080/15287390903578513 20391110

[43] Jones RR DellaValle CT Weyer PJ Robien K Cantor KP Krasner S. Ingested nitrate, disinfection by-products, and risk of colon and rectal cancers in the Iowa Women's Health Study cohort. Environ Int. (2019) 126:242–51. 10.1016/j.envint.2019.02.010 30822653 PMC10247223

[44] Bove GE Rogerson PA Vena JE. Case-control study of the effects of trihalomethanes on urinary bladder cancer risk. Arch Environ Occup Health. (2007) 62:39–47. 10.3200/AEOH.62.1.39-47 18171646

[45] Bove GE Rogerson PA Vena JE. Case control study of the geographic variability of exposure to disinfectant byproducts and risk for rectal cancer. Int J Health Geogr. (2007) 6:1–12. 10.1186/1476-072X-6-18 17535441 PMC1890278

[46] Chowdhury S Hall K. RETRACTED: human health risk assessment from exposure to trihalomethanes in Canadian cities. Environ Int. (2010) 36:453–60. 10.1016/j.envint.2010.04.001 20434775

[47] Krasner SW McGuire MJ Jacangelo JG Patania NL Reagan KM Aieta EM. The occurrence of disinfection by-products in US drinking water. J Am Water Works Assoc. (1989) 81:41–53. 10.1002/j.1551-8833.1989.tb03258.x

[48] Ding H Meng L Zhang H Yu J An W Hu J. Occurrence, profiling and prioritization of halogenated disinfection by-products in drinking water of China. Environ Sci. (2013) 15:1424–9. 10.1039/c300110e 23743579

[49] Christman RF Norwood DL Millington DS Johnson JD Stevens AA. Identity and yields of major halogenated products of aquatic fulvic acid chlorination. Environ Sci Technol. (1983) 17:625–8. 10.1021/es00116a012 22288709

[50] Quimby BD Delaney MF Uden PC Barnes RM. Determination of the aqueous chlorination products of humic substances by gas chromatography with microwave plasma emission detection. Anal Chem. (1980) 52:259–63. 10.1021/ac50052a010

[51] Stevens AA Moore LA Slocum CJ Smith BL Seeger DR Ireland JC. By-products of chlorination at ten operating utilities. Water Chlorin. (1990) 6:579–604.

[52] Blazak WF Meier JR Stewart BE Blachman DC Deahl JT. Activity of 1, 1, 1-and 1, 1, 3-trichloroacetones in a chromosomal aberration assay in CHO cells and the micronucleus and spermhead abnormality assays in mice. Mutat Res Genet Toxicol. (1988) 206:431–8. 10.1016/0165-1218(88)90050-X 3205262

[53] Merrick BA Smallwood CL Meier JR McKean DL Kaylor WH Condie LW. Chemical reactivity, cytotoxicity, and mutagenicity of chloropropanones. Toxicol Appl Pharmacol. (1987) 91:46–54. 10.1016/0041-008X(87)90192-X 3313810

[54] Xu X Mariano TM Laskin JD Weisel CP. Percutaneous absorption of trihalomethanes, haloacetic acids, and haloketones. Toxicol Appl Pharmacol. (2002) 184:19–26. 10.1006/taap.2002.9494 12392965

[55] Babaei AA Alavi N Hassani G Yousefian F Shirmardi M Atari L. Occurrence and related risk assessment of trihalomethanes in drinking water, Ahvaz, Iran. Fresenius Environ Bull. (2015) 24:4807–15.

[56] Mosaferi M Asadi M Aslani H Mohammadi A Abedi S Nemati Mansour S. Temporospatial variation and health risk assessment of trihalomethanes (THMs) in drinking water (northwest Iran). Environ Sci Pollut Res. (2021) 28:8168–80. 10.1007/s11356-020-11063-w 33052571

[57] Lee J Kim ES Roh BS Eom SW Zoh KD. Occurrence of disinfection by-products in tap water distribution systems and their associated health risk. Environ Monit Assess. (2013) 185:7675–91. 10.1007/s10661-013-3127-1 23446885

[58] Arman K PARDAKHTI A Osoleddini N Leili M. Cancer risk assessment from multi-exposure to chloroform in DrinkingWater of ilam city, Iran. (2016). 10.17795/ajehe-5331

[59] Ashley DL Blount BC Singer PC Depaz E Wilkes C Gordon S. Changes in blood trihalomethane concentrations resulting from differences in water quality and water use activities. Arch Environ Occup Health. (2005) 60:7–15. 10.3200/AEOH.60.1.7-15 16961003

[60] Haddad S Tardif GC Tardif R. Development of physiologically based toxicokinetic models for improving the human indoor exposure assessment to water contaminants: trichloroethylene and trihalomethanes. J Toxicol Environ Health Part A. (2006) 69:2095–136. 10.1080/15287390600631789 17060096

[61] Leavens TL Blount BC DeMarini DM Madden MC Valentine JL Case MW. Disposition of bromodichloromethane in humans following oral and dermal exposure. Toxicol Sci. (2007) 99:432–45. 10.1093/toxsci/kfm190 17656487

[62] Jo WK Weisel CP Lioy PJ. Routes of chloroform exposure and body burden from showering with chlorinated tap water. Risk Anal. (1990) 10:575–80. 10.1111/j.1539-6924.1990.tb00541.x 2287784

[63] Fryer AA Ramsay HM Lovatt TJ Jones PW Hawley CM Nicol DL. Polymorphisms in glutathione S-transferases and non-melanoma skin cancer risk in Australian renal transplant recipients. Carcinogenesis. (2005) 26:185–91. 10.1093/carcin/bgh291 15459020

[64] DeMarini DM Shelton ML Warren SH Ross TM Shim JY Richard AM. Glutathione S-transferase-mediated induction of GC AT transitions by halomethanes in salmonella. Environ Mol Mutagen. (1997) 30:440–7. 10.1002/(SICI)1098-2280(1997)30:4<440::AID-EM9>3.0.CO;2-M 9435885

[65] Landi S Hanley NM Kligerman AD DeMarini DM. Induction of sister chromatid exchanges in human peripheral blood lymphocytes by bromoform: investigation of the role of GSTT1-1 polymorphism. Mutat Res. (1999) 429:261–7. 10.1016/S0027-5107(99)00107-4 10526210

[66] Landi S Hanley NM Warren SH Pegram RA DeMarini DM. Induction of genetic damage in human lymphocytes and mutations in Salmonella by trihalomethanes: role of red blood cells and GSTT1-1 polymorphism. Mutagenesis. (1999) 14:479–82. 10.1093/mutage/14.5.479 10473651

[67] Landi S Naccarati A Ross MK Hanley NM Dailey L Devlin RB. Induction of DNA strand breaks by trihalomethanes in primary human lung epithelial cells. Mutat Res. (2003) 538:41–50. 10.1016/S1383-5718(03)00086-X 12834753

[68] Pegram RA Andersen ME Warren SH Ross TM Claxton LD. GlutathioneS-transferase-mediated mutagenicity of trihalomethanes in *Salmonella typhimurium*: contrasting results with bromodichloromethane and chloroform. Toxicol Appl Pharmacol. (1997) 144:183–8. 10.1006/taap.1997.8123 9169083

[69] Lilly PD Simmons JE Pegram RA. Dose-dependent vehicle differences in the acute toxicity of bromodichloromethane. Toxicol Sci. (1994) 23:132–40. 10.1093/toxsci/23.1.132 7958557

[70] Geter DR Chang LW Hanley NM Ross MK Pegram RA DeAngelo AB. Analysis of *in vivo* and *in vitro* DNA strand breaks from trihalomethane exposure. J Carcinog. (2004) 3:2. 10.1186/1477-3163-3-2 14969591 PMC395841

[71] Ross MK Pegram RA. *In vitro* biotransformation and genotoxicity of the drinking water disinfection byproduct bromodichloromethane: DNA binding mediated by glutathione transferase theta 1-1. Toxicol Appl Pharmacol. (2004) 195:166–81. 10.1016/j.taap.2003.11.019 14998683

[72] National Toxicology Program. Toxicology Studies of Bromodichloromethane (CAS No. 75-27-4) in Genetically Modified (FVB Tg. AC Hemizygous) Mice (Dermal, Drinking Water, and Gavage Studies) and Carcinogenicity Studies of Bromodichloromethane in Genetically Modified [B6. 129-Trp53 (tm1Brd)(N5) Haploinsufficient] Mice (Drinking Water and Gavage Studies). National Toxicology Program Genetically Modified Model Report (2007). p. 1. PMC8950459 18784761

[73] Richardson SD DeMarini DM Kogevinas M Fernandez P Marco E Lourencetti C. What's in the pool? A comprehensive identification of disinfection by-products and assessment of mutagenicity of chlorinated and brominated swimming pool water. Environ Health Perspect. (2010) 118:1523–30. 10.1289/ehp.1001965 20833605 PMC2974688

[74] Plewa MJ Wagner ED Mitch WA. Comparative mammalian cell cytotoxicity of water concentrates from disinfected recreational pools. Environ Sci Technol. (2011) 45:4159–65. 10.1021/es104284h 21466188

[75] Wang D Xu Z Zhao Y Yan X Shi J. Change of genotoxicity for raw and finished water: role of purification processes. Chemosphere. (2011) 83:14–20. 10.1016/j.chemosphere.2011.01.039 21315407

[76] Stalter D Dutt M Escher BI. Headspace-free setup of *in vitro* bioassays for the evaluation of volatile disinfection by-products. Chem Res Toxicol. (2013) 26:1605–14. 10.1021/tx400263h 24117097

[77] Faustino-Rocha AI Rodrigues D da Costa RG Diniz C Arag ao S Talhada D. Trihalomethanes in liver pathology: mitochondrial dysfunction and oxidative stress in the mouse. Environ Toxicol. (2016) 31:1009–16. 10.1002/tox.22110 25640707

[78] Fisher D Yonkos L Ziegler G Friedel E Burton D. Acute and chronic toxicity of selected disinfection byproducts to *Daphnia magna, Cyprinodon variegatus*, and *Isochrysis galbana*. Water Res. (2014) 55:233–44. 10.1016/j.watres.2014.01.056 24607524

[79] Pagé-Larivière F Tremblay A Campagna C Rodriguez MJ Sirard MA. Low concentrations of bromodichloromethane induce a toxicogenomic response in porcine embryos *in vitro*. Reprod Toxicol. (2016) 66:44–55. 10.1016/j.reprotox.2016.09.010 27671623

[80] Kogevinas M Villanueva CM Font-Ribera L Liviac D Bustamante M Espinoza F. Genotoxic effects in swimmers exposed to disinfection by-products in indoor swimming pools. Environ Health Perspect. (2010) 118:1531–7. 10.1289/ehp.1001959 20833606 PMC2974689

[81] Cantor KP Villanueva CM Silverman DT Figueroa JD Real FX Garcia-Closas M. Polymorphisms in GSTT1, GSTZ1, and CYP2E1, disinfection by-products, and risk of bladder cancer in Spain. Environ Health Perspect. (2010) 118:1545–50. 10.1289/ehp.1002206 20675267 PMC2974691

[82] Kargalioglu Y McMillan BJ Minear RA Plewa MJ. Analysis of the cytotoxicity and mutagenicity of drinking water disinfection by-products in *Salmonella typhimurium*. Teratog Carcinog Mutag. (2002) 22:113–28. 10.1002/tcm.10010 11835289

[83] Parinet J Tabaries S Coulomb B Vassalo L Boudenne JL. Exposure levels to brominated compounds in seawater swimming pools treated with chlorine. Water Res. (2012) 46:828–36. 10.1016/j.watres.2011.11.060 22153961

[84] Thier R Taylor JB Pemble SE Humphreys WG Persmark M Ketterer B. Expression of mammalian glutathione S-transferase 5-5 in Salmonella typhimurium TA1535 leads to base-pair mutations upon exposure to dihalomethanes. Proc Natl Acad Sci USA. (1993) 90:8576–80. 10.1073/pnas.90.18.8576 8378332 PMC47400

[85] Geter DR George MH Moore TM Kilburn S Huggins-Clark G DeAngelo AB. Vehicle and mode of administration effects on the induction of aberrant crypt foci in the colons of male F344/N rats exposed to bromodichloromethane. J Toxicol Environ Health Part A. (2004) 67:23–9. 10.1080/15287390490253642 14668109

[86] Fujie K Aoki T Wada M. Acute and subacute cytogenetic effects of the trihalomethanes on rat bone marrow cells *in vivo*. Mutat Res. (1990) 242:111–9. 10.1016/0165-1218(90)90036-2 2233827

[87] Richardson SD Plewa MJ Wagner ED Schoeny R DeMarini DM. Occurrence, genotoxicity, and carcinogenicity of regulated and emerging disinfection by-products in drinking water: a review and roadmap for research. Mutat Res. (2007) 636:178–242. 10.1016/j.mrrev.2007.09.001 17980649

[88] Kundu B Richardson SD Granville CA Shaughnessy DT Hanley NM Swartz PD. Comparative mutagenicity of halomethanes and halonitromethanes in Salmonella TA100: structure-activity analysis and mutation spectra. Mutat Res. (2004) 554:335–50. 10.1016/j.mrfmmm.2004.05.015 15450430

[89] DeAngelo AB Geter DR Rosenberg DW Crary CK George MH. The induction of aberrant crypt foci (ACF) in the colons of rats by trihalomethanes administered in the drinking water. Cancer Lett. (2002) 187:25–31. 10.1016/S0304-3835(02)00356-7 12359347

[90] Ming ME Levy RM Hoffstad OJ Filip J Gimotty PA Margolis DJ. Validity of patient self-reported history of skin cancer. Arch Dermatol. (2004) 140:730–5. 10.1001/archderm.140.6.730 15210466

